# *z*Trap: zebrafish gene trap and enhancer trap database

**DOI:** 10.1186/1471-213X-10-105

**Published:** 2010-10-18

**Authors:** Koichi Kawakami, Gembu Abe, Tokuko Asada, Kazuhide Asakawa, Ryuichi Fukuda, Aki Ito, Pradeep Lal, Naoko Mouri, Akira Muto, Maximilliano L Suster, Hitomi Takakubo, Akihiro Urasaki, Hironori Wada, Mikio Yoshida

**Affiliations:** 1 Division of Molecular and Developmental Biology, National Institute of Genetics, 1111 Yata, Mishima, Shizuoka 411-8540, Japan; 2 Department of Genetics, Graduate University for Advanced Studies (SOKENDAI), 1111 Yata, Mishima, Shizuoka 411-8540, Japan; 3 PRESTO, Japan Science and Technology Agency (JST), Honcho 4-1-8, Kawaguchi, Saitama 322-0012, Japan; 4 Intec Systems Institute Inc., 1-3-3 Shinsuna, Koto-ku, Tokyo 136-0075, Japan; 5 Current Address: Sars International Centre for Marine Molecular Biology, Thormøhlensgate 55, N-5008 Bergen, Norway

## Abstract

**Background:**

We have developed genetic methods in zebrafish by using the *Tol2 *transposable element; namely, transgenesis, gene trapping, enhancer trapping and the Gal4FF-UAS system. Gene trap constructs contain a splice acceptor and the GFP or Gal4FF (a modified version of the yeast Gal4 transcription activator) gene, and enhancer trap constructs contain the zebrafish *hsp70l *promoter and the GFP or Gal4FF gene. By performing genetic screens using these constructs, we have generated transgenic zebrafish that express GFP and Gal4FF in specific cells, tissues and organs. Gal4FF expression is visualized by creating double transgenic fish carrying a Gal4FF transgene and the GFP reporter gene placed downstream of the Gal4-recognition sequence (UAS). Further, the Gal4FF-expressing cells can be manipulated by mating with UAS effector fish. For instance, when fish expressing Gal4FF in specific neurons are crossed with the UAS:TeTxLC fish carrying the tetanus neurotoxin gene downstream of UAS, the neuronal activities are inhibited in the double transgenic fish. Thus, these transgenic fish are useful to study developmental biology and neurobiology.

**Description:**

To increase the usefulness of the transgenic fish resource, we developed a web-based database named *z*Trap http://kawakami.lab.nig.ac.jp/ztrap/. The *z*Trap database contains images of GFP and Gal4FF expression patterns, and genomic DNA sequences surrounding the integration sites of the gene trap and enhancer trap constructs. The integration sites are mapped onto the *Ensembl *zebrafish genome by in-house Blat analysis and can be viewed on the *z*Trap and *Ensembl *genome browsers. Furthermore, *z*Trap is equipped with the functionality to search these data for expression patterns and genomic loci of interest. *z*Trap contains the information about transgenic fish including UAS reporter and effector fish.

**Conclusion:**

*z*Trap is a useful resource to find gene trap and enhancer trap fish lines that express GFP and Gal4FF in desired patterns, and to find insertions of the gene trap and enhancer trap constructs that are located within or near genes of interest. These transgenic fish can be utilized to observe specific cell types during embryogenesis, to manipulate their functions, and to discover novel genes and *cis*-regulatory elements. Therefore, *z*Trap should facilitate studies on genomics, developmental biology and neurobiology utilizing the transgenic zebrafish resource.

## Background

Zebrafish has been used as a model vertebrate because of high fecundity, rapid embryonic development, transparency during embryonic stages and inexpensive and easy breeding. We have developed a transposon technology by using the medaka fish *Tol2 *transposable element in this model vertebrate [1-5]. With the *Tol2 *transposon technology, it is now possible for researchers to perform highly efficient transgenesis in zebrafish and powerful genetic approaches such as gene trapping, enhancer trapping and targeted gene expression by the Gal4-UAS system [4,6-10]. These have greatly advanced genetic studies in zebrafish and increased the usefulness of zebrafish as a vertebrate model.

**Figure 1:**
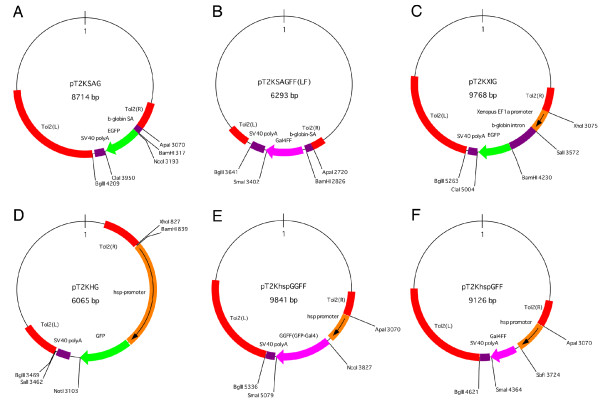
***Tol2 *transposon constructs used to create transgenic zebrafish**. (A) The T2KSAG gene trap construct [4]. (B) The T2KSAGFF(LF) gene trap construct [10]. LF in parentheses stands for one loxP and one FRT site embedded in the construct. (C) The T2KXIG construct [4]. (D) The T2KHG enhancer trap construct [9]. (E) The T2KhspGFF enhancer trap construct [10]. (F) The T2KhspGFF enhancer trap construct [10]. Red: *Tol2 *sequence. Green: EGFP. Orange: promoter. Purple: splice acceptor, poly A signal and intron. Magenta: Gal4FF and GFP-Gal4FF fusion.

We have constructed different types of *Tol2*-based gene trap and enhancer trap constructs. T2KSAG and T2KSAGFF (referred to as T2KSAGFF(LF) hereafter) are gene trap constructs that contain the GFP and Gal4FF gene, a modified version of the yeast Gal4 transcription activator, downstream of the rabbit β-globin splice acceptor (SA), respectively. T2KHG, T2KhspGGFF and T2KhspGFF are enhancer trap constructs that contain the GFP, Gal4FF-GFP fusion, and Gal4FF gene downstream of the zebrafish *hsp70l *promoter, respectively [4,9,10] (Figure 1). To visualize Gal4FF expression, we constructed transgenic fish carrying the GFP or RFP reporter gene downstream of the Gal4 recognition sequence (UAS:GFP and UAS:RFP). By using these constructs and transgenic fish, we have performed genetic screens, and generated a large number of transgenic fish that express GFP and Gal4FF in specific tissues, cells and organs.

These transgenic fish have been useful for various studies in developmental biology, genomics and neurobiology. For example, a novel *hoxC *transcript was revealed by the SAGp22A line [4], new functions for the *tcf7 *gene and the *synembryn-like *gene were uncovered using HG21C and HGn8H [9], mitochondrion-rich cells on the skin were visualized using HG9B [11], dilatation of the cardiac ventricle was analyzed using SAG4A [12], the *misty somites *gene encoding a maternal factor involved in somitogenesis was discovered in SAG20A [13,14], innervation of hair cells by afferent neurons was visualized in HGn39D [15], and nasal-temporal patterning of the retina by Fgf signaling was demonstrated using HGn42A [16]. More recent examples include studies of cell-fate determination in the notochord using SAGFF214A [17], characterization of the somatotopic projections of the lateral line afferent neurons in the CNS using hspGFF53A [18], sex-reversal in the *fancl *mutant HG10A [19], patterning of the lymphatic system (SAGFF27C;[20]), and lens fiber differentiation (SAGFF168A;[21]). Furthermore, the Gal4FF-expressing transgenic fish can be used for targeted expression of a desired gene in a desired place. For instance, we created transgenic fish carrying the tetanus neurotoxin gene downstream of the Gal4 recognition sequence (UAS:TeTxLC and UAS:TeTxLC:CFP). When these UAS effector fish were crossed with transgenic fish that expressed Gal4FF in subsets of the spinal neurons or olfactory neurons (SAGFF31B, SAGFF36B, and SAGFF27A), double transgenic fish exhibited specific behavioral abnormalities, demonstrating successful inhibition of the specific neural circuits by targeted expression of the tetanus toxin [10,22].

Previously, GFP enhancer trap screens using *Tol2 *[6] or *Sleeping Beauty *[23], an enhancer trap screen by using a retroviral vector [24,25], gene trap and enhancer trap screens to generate transgenic fish that express Gal4 in specific patterns [7,8] were carried out also in other laboratories. Although a database containing the data from 27 GFP-expressing fish was created previously [26]http://plover.imcb.a-star.edu.sg/~zetrap/ZETRAP.htm, it is at present difficult to search most of these transgenic fish because of the absence of searchable databases. To make the transgenic fish generated in our laboratory more useful, we aimed to develop a database equipped with search functions that contains the expression pattern data and the integration site data from our transgenic fish. Here we report a web-based database named *z*Trap (zebrafish gene trap and enhancer trap database).

## Construction and content

**Figure 2:**
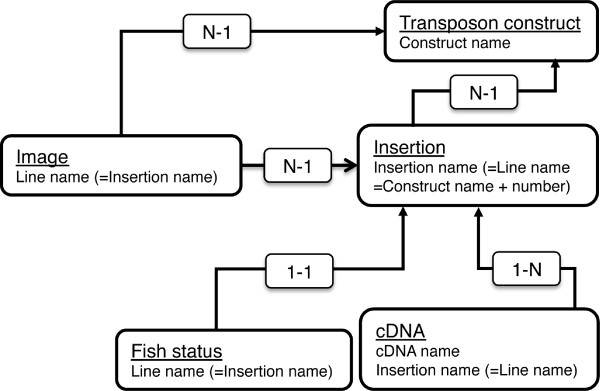
**The zTrap database scheme.**  The image, insertion, cDNA and fish status data are connected via line names, which are  the same as insertion names. A line name (insertion name) is composed of a construct  name, number and a letter. The transposon construct data are connected via the construct  name. “N-1” and “1-1” denote many-to-one and one-to-one relationships, respectively.  An instance of the N-1 relationship is that two images are linked with the SAG2A fish  line (insertion) in Figure 4A.

The *z*Trap database was constructed by using the following applications: OS, Red Hat Enterprise Linux; the database management system, MySQL 5.0; the application server, Tomcat 5.5; the user interface, Java Server Face 1. 1; the web server, Apache 2. The *z*Trap database scheme is depicted in Figure 2. The database contains image, insertion, cDNA, transposon construct, and fish status data. The image data have a line name, which corresponds to the name of the insertion carried by the transgenic line, and thus the image and insertion data are connected. Also, the line name is used to connect these data to the fish status and cDNA data. A line name consists of transposon construct name, number and letter, and thereby the image and insertion data are connected to the transposon construct data.

**Figure 3:**
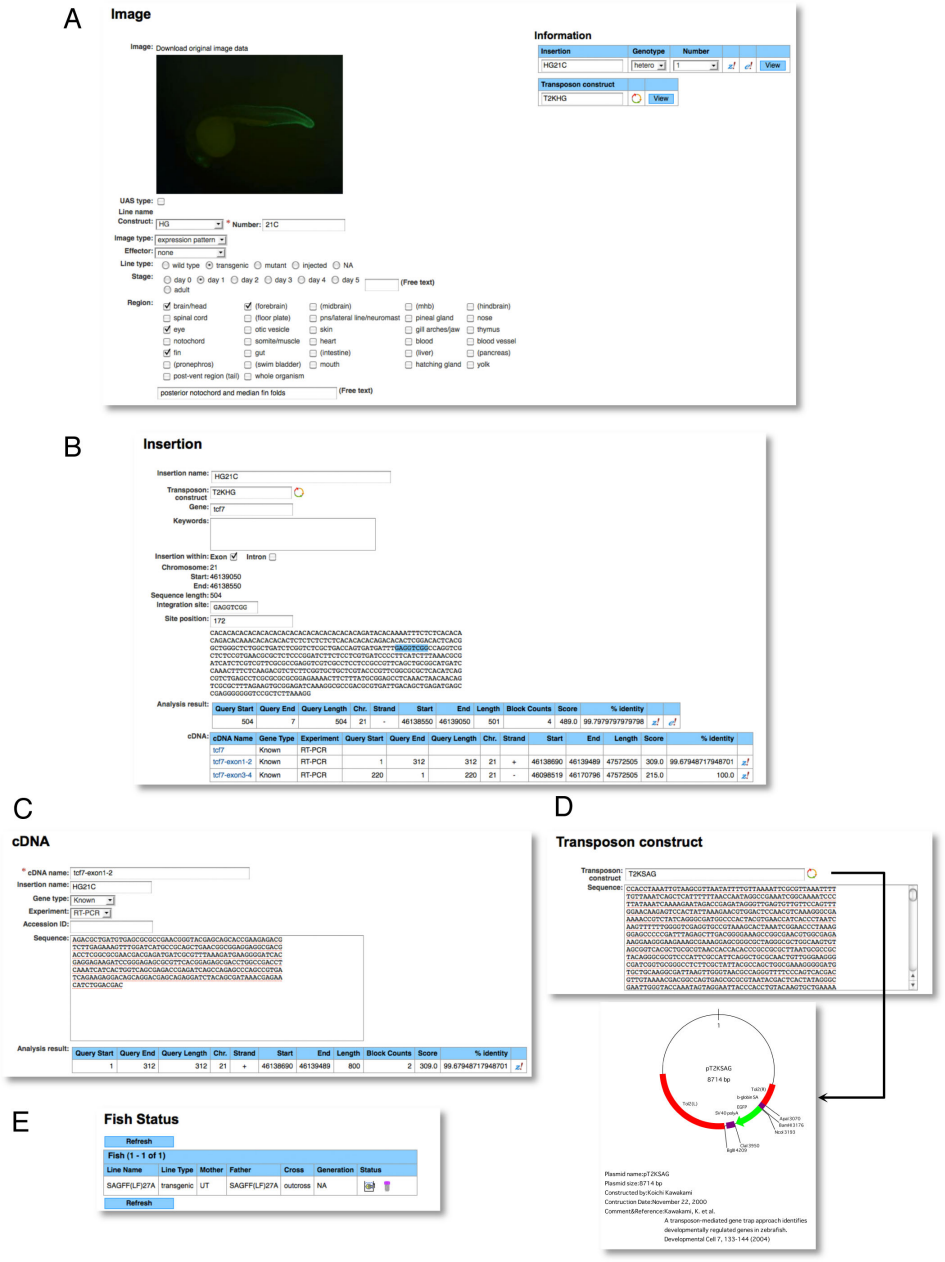
**The content of the *z*Trap database**. (A) The image data form of the HG21C transgenic fish [9] is shown as an example. A GFP fluorescence image at 1 dpf and regions showing GFP expression (forebrain, eye and fin fold) are described. The image data have links to the insertion data and the transposon construct data. (B) The insertion data form of the HG21C transgenic fish. 504-bp DNA sequence surrounding the integration site which is mapped on the chromosome 21 is shown. An 8-bp sequence which is duplicated upon integration is highlighted in blue. The data have links to the transposon construct data and cDNA data and the *z! *and *e! *genome browsers. (C) The cDNA data form of the *tcf7 *transcript that has a link to the HG21C insertion data. (D) The transposon construct data form of pT2KSAG. The data contain the sequence and map. (E) The fish status data form of the SAGFF(LF)27A. The fish tank and tube icons mean this line is kept alive and as a frozen sperm, respectively.

(1) Image data: we have performed gene trap and enhancer trap screens and created transgenic fish that expressed GFP and Gal4FF in spatially and temporally restricted patterns. Image data were generated from these transgenic fish lines. The image data consist of image; line name (construct plus number); image type (selected from expression pattern, in situ or movie); effector (UAS lines used to visualize Gal4FF expression); line type (for now, transgenic only); stage (when the image was taken); region (where the expression was observed); and information (links to insertion and transposon construct data) (Figure 3A).

Images are acquired by photographing transgenic embryos with a CCD camera under a fluorescent stereo microscope. When we obtain an image of the offspring from fish injected with a *Tol2 *construct, we first assign a line name to the image data. The line name is composed of an abbreviation of the *Tol2 *construct used (namely, SAG for T2KSAG, HG for T2KHG, hspGGFF for T2KhspGGFF, hspGFF for T2KhspGFF, and SAGFF(LF) for T2KSAGFF(LF)), a number given to individual founder fish in the order of successful mating, and a letter that discriminates different expression patterns observed in offspring from the same founder. To describe the GFP expression patterns, we selected 32 terms from anatomical ontology in ZFIN http://zfin.org/action/anatomy/search. By using these terms, expression annotation is carried out in our weekly screen meetings. New anatomical terms may be added to the data sheet when new expression patterns are observed. Currently, the image data were created for previously published transgenic lines; namely, 37 SAG lines [4], 72 HG lines [9], 28 hspGGFF lines [10], 1 hspGFF line [18], and 9 SAGFF(LF) lines [10,17,20-22], and more data for new transgenic fish are being added daily.

**Figure 4:**
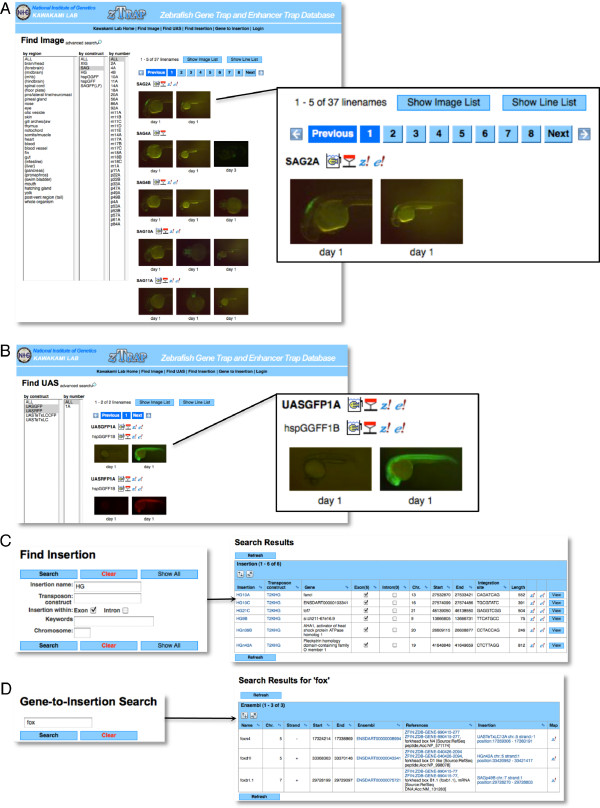
**The functionality to search fish and insertions**. (A) "Find image" is a main page to find gene trap and enhancer trap fish with desired patterns. Thumbnails of images of fish lines appear when an expression pattern and/or construct name is selected in the "by region", "by construct" and "by number" columns on the left. Here, ALL in "by region", SAG in "by construct" and ALL in "by number" are chosen, and all "SAG" transgenic fish are seen on the right. These can be seen also as a list style by clicking "Show Image List" or "Show Line List". Icons next to the line name link to the fish status data, the insertion data and the *z! *and *e! *genome browsers. (B) "Find UAS" allows users to find transgenic fish carrying a reporter or an effector gene downstream of the Gal4 recognition sequence. Here, double transgenic fish carrying the hspGGFF1B insertion and UASGFP or UASRFP are shown. Icons next to the line names link to the fish status data, the insertion data and the *z! *and *e! *genome browsers. (C) "Find insertion" allows users to search the insertion data by filling in the blanks. Here, "HG" is typed in "insertion name" and the "exon" checkbox is checked, and all insertions that are created by using the T2KHG construct and located within an exon are seen. (D) "Gene-to-Insertion Search" allows users to find insertions located near a gene of interest. Here, "fox" is typed, and all insertions located within a distance of 100-kb from the *fox *genes are picked up.

*z*Trap contains image data for UAS reporter and effector lines and other transgenic lines as well; i.e., the UASGFP, UASRFP, UASTeTxLC, UASTeTxLCCFP, and XIG lines. These transgenic fish were created in our laboratory also by *Tol2*-mediated transgensis. Image data of UAS lines are acquired by crossing these lines with appropriate Gal4 drivers. The information of the Gal4 driver is also attached to the image data of the UAS lines (Figure 4B). The XIG fish carries the GFP gene under the control of the EF1α promoter [4]. For UASTeTxLCCFP, movie files were uploaded to show behavioral defects in double transgenic fish more clearly.

(2) Insertion data: all of transgenic fish created in our laboratory have been analyzed by Southern blot hybridization to identify fish with single *Tol2 *insertions. When fish turn out to carry multiple insertions, they are mated with non-transgenic fish to generate fish with single insertions in the next generation [27]. This process is important to exclude ambiguity concerning the relationship between an expression pattern and a causative insertion, and also makes it easy to amplify genomic DNA surrounding the *Tol2 *insertions by inverse PCR and adaptor-ligation PCR. Insertion data are created based on these analyses.

The insertion data consist of the insertion name (which corresponds to a line name); a transposon construct used to create the insertion; gene (when *Tol2 *is integrated within a gene); keywords (given manually); checkboxes to show whether it is located within exon or intron; its chromosomal position; genomic DNA sequence at the integration site retrieved from inverse PCR or adaptor-ligation PCR; and the result of in-house Blat analysis using the DNA sequence. The position of the 8-bp target site duplication created upon integration is highlighted in blue in the DNA sequence (Figure 3B).

The genomic DNA sequence surrounding the integration site is analyzed by in-house Blat search against the *Danio rerio *genomic data that is downloaded from the *Ensembl *FTP server ftp://ftp.ensembl.org/pub/current_fasta/danio_rerio/dna/. Currently Zv8 is used, and a newer version of the genome data will be downloaded when it becomes available. When the integration site is successfully mapped on the *Ensembl *genome, the chromosome number and the start and end positions are incorporated into the insertion data. The mapping process is in part carried out manually when the in-house Blat analysis showed no hit or multiple hits. If the integration site is mapped within an annotated transcript, the gene name and specific location within an exon or intron are added manually later. Keywords are added also manually. If transcripts surrounding the insertion are analyzed by cDNA cloning, the cDNA data are linked to the corresponding insertion data. The insertion data have been created for all of 152 transgenic lines described above, and more data are being added daily.

(3) cDNA data: when we analyzed transcripts trapped by gene trap insertions or located near integration loci by RT-PCR, 5'RACE, 3'RACE or other methods, cDNA data are created. The cDNA data contain the cDNA name, the corresponding insertion name, a gene type (known, unknown or predicted), a type of experiment (RT-PCR, 5'RACE or 3'RACE), sequence itself and the result of in-house Blat analysis using the sequence (Figure 3C). This analysis is optional and is not carried out for all transgenic lines. Of the 147 insertions of the gene trap and enhancer trap constructs described above, 20 were analyzed for cDNA data.

(4) Transposon construct data: these data contain the restriction map and sequence information of the transposon constructs used to create the transgenic fish (Figure 3D). Currently, these include the gene trap and enhancer trap constructs (T2KSAG, T2KSAGFF(LF), T2KHG, T2KhspGGFF, and T2KhspGFF), the T2KXIG construct (Figure 1), and the UAS reporter and effector constructs (UAS:GFP, UAS:RFP, and UAS:TeTxLC:CFP).

(5) Fish status data: these data indicate the status of transgenic fish in our laboratory; i.e., whether they are kept alive in the fish room (a "fish tank" icon) stored as frozen sperms (a "tube" icon), or whether they have been terminated or lost (a "fish skeleton" icon)(Figure 3E).

## Utility and Discussion

### Find image

The "Find Image" page is the main page of the *z*Trap database (Figure 4A and additional file 1). From this page, users can search the database by clicking a term in the "by region" column, a construct name in the "by construct" column and a number in the "by number" column, and then thumbnail images of corresponding transgenic lines will appear on the right. Thus, users can find transgenic fish lines that show expression patterns of interest or that are created by using a construct of interest. In these columns, "ALL" can also be selected. The "advanced search" allows users to select regions and stages with more flexibility. When the thumbnail image is clicked, the image data will open. To view many images at the same time, the image data can be shown as a list by clicking "Show Image List" or "Show Line List" (Figure 4A).

There are four types of icons next to the line name. A "fish tank" icon opens the fish status data. These data are useful when users request transgenic fish from us. A "transposon icon" (depicted in a shape of a inverted triangle) opens the corresponding insertion data (Figure 4A). "*z*!" and "*e*!" icons link with the *z*Trap and *Ensembl *genome browsers, respectively, and appear next to the line name when the genomic DNA sequence at the integration site was successfully mapped onto the *Ensembl *genome by in-house Blat analysis.

### Find UAS

The UAS-reporter and UAS-effector fish that carry a reporter or effector gene downstream of the Gal4 recognition sequence are useful to visualize and manipulate Gal4FF-expressing cells. Such UAS transgenic fish can be explored by clicking "Find UAS" on the menu bar (Figure 4B). The "Find UAS" page has a similar function to that of the "Find Image" page but lacks the "by region" column since it is not applicable to the UAS fish. UAS reporter or effector fish of interest are easily found by clicking a construct in the "by construct" column. Thumbnail images (in some cases, movies) of the UAS fish, the line names, icons, and the information on Gal4FF drivers will appear on the right. We also analyzed the integration sites of the UAS constructs by inverse PCR and adaptor-ligation PCR. Thus, insertion data are created for all of the UAS fish lines and linked to the UAS image data.

### Find Insertion

"Find Insertion" on the menu bar opens dialog boxes to search insertion data by an insertion name, a transposon construct name, checkboxes ("Exon" and "Intron"), keywords, and a chromosome number (Figure 4C). The corresponding insertion data will be shown as a list. From the list, it is possible to view the insertion site and jump to the insertion data and the genome browsers. The "Exon" and "Intron" checkboxes are useful to find possible insertional mutations caused by integration of a *Tol2 *construct. These two checkboxes can be checked simultaneously. Search by the chromosome number is useful to identify insertions located near a locus of interest. Such insertions may be used for genetic mapping or as dominant markers on a balancer chromosome to maintain a lethal mutation.

### Gene-to-Insertion Search

We developed the "Gene-to-Insertion" program to further facilitate searching the insertion data. "Gene-to-Insertion" on the menu bar opens a dialog box (Figure 4D). Users can type a gene name (*hox*, *sox*, etc.), gene ID starting from *zgc *(*ZFIN *gene name) or ENSDARG (*Ensembl *genes), or any words linked to the *Ensembl *transcript database. The Gene-to-Insertion program first identifies genes that have the typed word in their descriptions, and then finds insertions that are located within a distance of 100-kb from the identified genes. Thus, users may find transgenic fish lines that express GFP or Gal4FF in a pattern that corresponds to that of a gene of interest or may find an insertional mutation of a gene of interest.

### The *z*Trap genome browser

**Figure 5:**
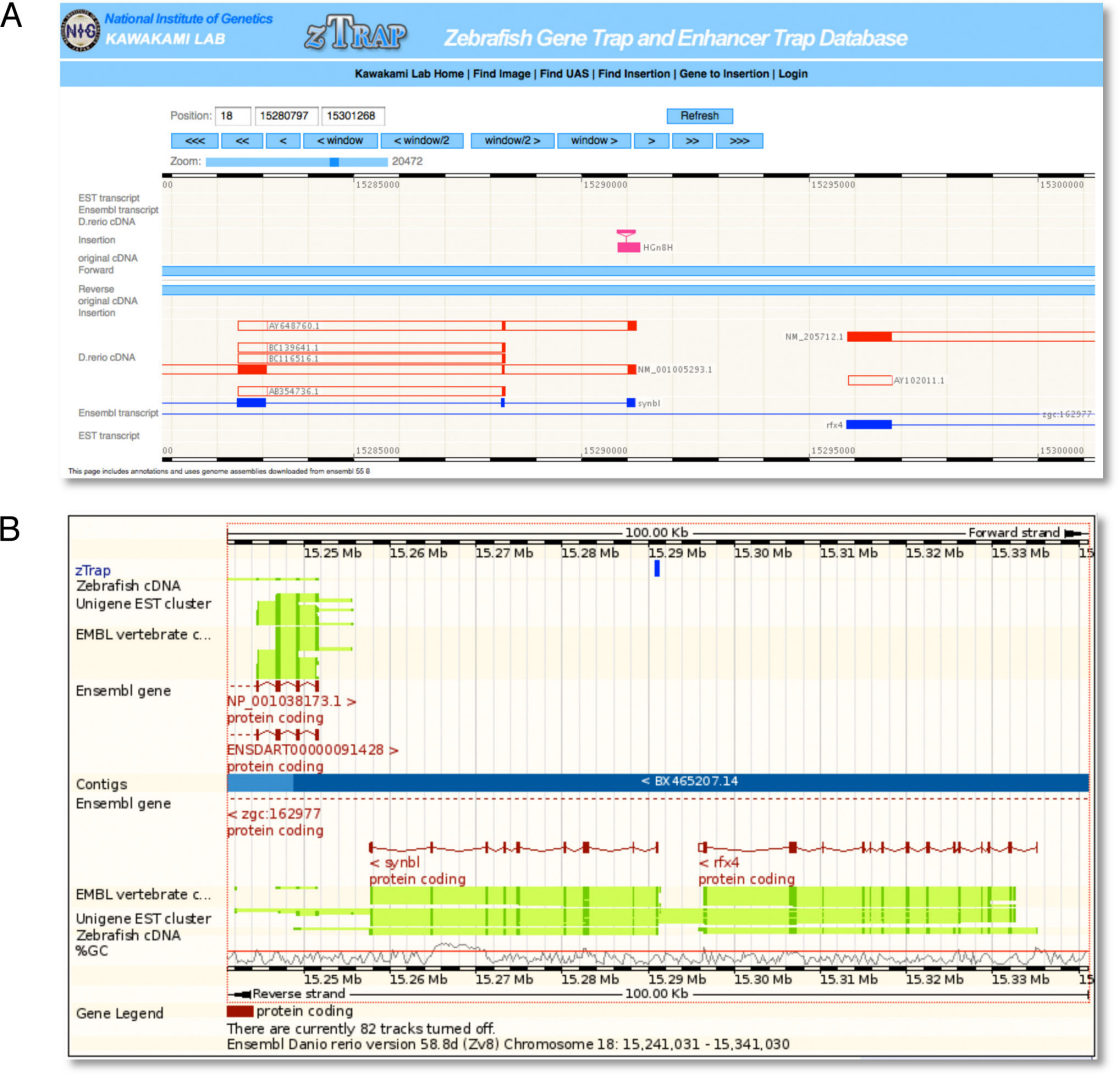
**Visualization of insertions on genome browsers**. (A) The *z*Trap genome browser showing a view surrounding the HG21C insertion. The HG21C insertion (a pink bar) is located within the first exon of the *tcf7 *gene [9] that are represented by multiple tracks below forward and reverse genomic sequences (two blue lines in the center), such as *D. rerio *cDNA, *Ensembl *transcripts and EST transcripts. Note that transcripts above the genomic sequences go from left to right and transcripts below them go in the opposite direction. (B) The *Ensembl *genome browser showing the HGn8H insertion [9]. A track for *z*Trap insertions can be attached on the *Ensembl *genome browser as follows; (1) from the "region in detail" display page, select "configure this page" on the side bar, (2) select "custom data" on the top bar, (3) select "Attach DAS", (4) type "ztrap" in the "Filter sources" box and click "Next", (5) check the "zTrap" check box, click "Next", and close the pop-up window.

In order to see the genomic landscape surrounding the integration site quickly, we developed the *z*Trap genome browser. The "*z!*" icon found in many places is used to open the browser (Figure 5A). On the *z*Trap genome browser, 100-kb of the genomic sequence surrounding a *Tol2 *insertion (indicated by a pink bar with an inverted triangle) can be viewed with zoom-in and zoom-out functions. The *Tol2 *construct can be inserted in two different orientations. We previously defined the left (L) and right (R) ends of *Tol2 *with respect to the direction of the transposase gene [28]. When the insertion is placed above the genomic sequence (two blue lines in the center), the L end of *Tol2 *is located on the left, and when the insertion is placed below the genomic sequence, the L end is located on the right. The *z! *browser essentially contains the same data as the *Ensembl *browser. The *D. rerio *cDNA, *Ensembl *transcript and EST transcript data are downloaded from *Ensembl *and can be seen on multiple tracks of the *z! *browser. Descriptions about the insertion, *D. rerio *cDNA, *Ensembl *transcript and EST transcript appear as pop-ups when they are clicked. In addition, other insertions and original cDNA located within a distance of 100-kb can be seen simultaneously.

### Link to *Ensembl*

When the "*e*!" icon found in many places is clicked, the positional information of the integration site is transferred to the *Ensembl *genome browser, and the "region in detail" display of the corresponding genomic locus will open. Furthermore, we developed a DAS (Distributed Annotation System) server. The *z*Trap track appears on the *Ensembl *genome browser by executing the following procedure (Figure 5B); (1) from the "region in detail" display page, select "configure this page", (2) select "custom data" on the top bar, (3) select "Attach DAS", (4) type "ztrap" in the "Filter sources" box and click "Next", (5) check the "zTrap" check box and click "Next", and close the pop-up window.

## Conclusions

The *z*Trap database developed in this study allows users to rapidly search a large number of transgenic fish lines on two major criteria: i.e., expression patterns of interest and genes of interest. Using these search functions, researchers may find transgenic fish that express GFP or Gal4FF in desired patterns which can be used in several ways. First, transgenic fish may serve as live markers for studies of cell proliferation, differentiation and migration during embryogenesis and organogenesis. Second, transgenic fish expressing Gal4FF may be used to manipulate specific cell types in combination with appropriate UAS-effector fish. Third, by analyzing the genomic locus surrounding the integration site and associated transcripts, a novel gene, transcript and/or *cis*-regulatory elements responsible for the expression pattern may be discovered. Finally, it is possible that the transposon insertion could disrupt the function of a gene of interest or a previously uncharacterized gene. Altogether, the *z*Trap database and our transgenic zebrafish resources should facilitate studies on genomics, developmental biology and neurobiology.

## Availability and requirements

The *z*Trap database is publicly accessible at http://kawakami.lab.nig.ac.jp/ztrap/. Browsers recommended are: Firefox 2 or later; Internet Explorer 6 or later; Safari 2 or later. The copy and use of the content (text, graphics, images and other materials) are permitted with prior agreement from the corresponding author.

## Authors' contributions

KK designed the database and wrote the manuscript. GA, TA, KA, RF, AI, PL, NM, AM, MLS, HT, AU and HW created the data. MY designed and constructed the database. All authors read and approved the manuscript.

## Supplementary Material

Additional file 1 ***z*Trap database navigation**. A figure that shows how to jump to the contents from "Find image" page. Click here for file
