# Supplementary material for: zTrap: zebrafish gene trap and enhancer trap database
Source: BMC Dev Biol. 2010 Oct 18;10:105. doi: 10.1186/1471-213X-10-105 (PMC2970601; doi:10.1186/1471-213X-10-105)
Supplement: Additional file 1 — zTrap database navigation. A figure that shows how to jump to the contents from "Find image" page. [file 1471-213X-10-105-S1.PDF]

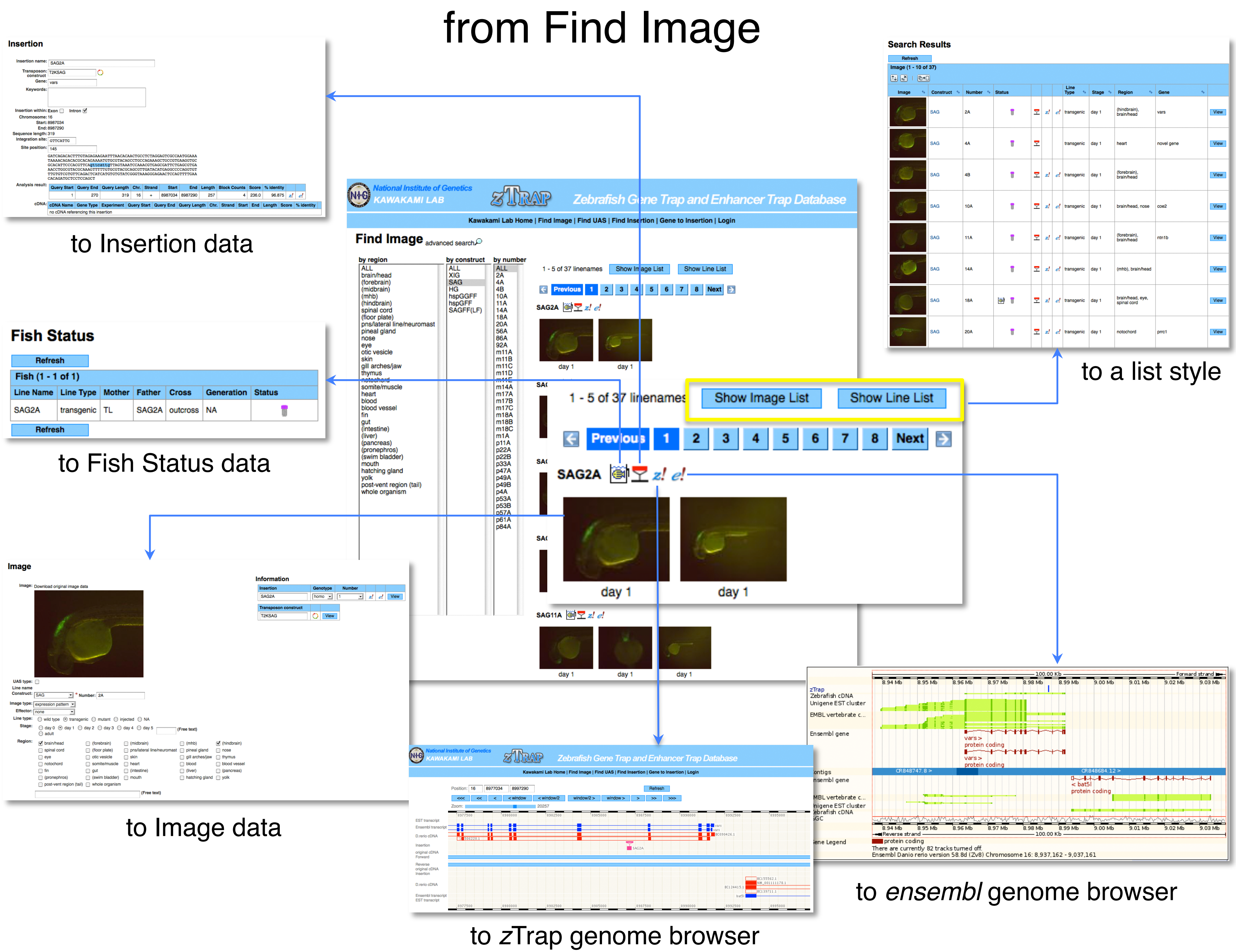
from Find Image
to Insertion data
to Fish Status data
to Image data
to zTrap genome browser
to ensembl genome browser
to a list style
Show Image List
Show Line List
1 - 5 of 37 linenames
Previous
Next
SAG2A
day 1
Fish Status
Find Image
Search Results
Insertion
Image
Zebrafish Gene Trap and Enhancer Trap Database
